# Supplementary material for: Soil nitrogen concentration mediates the relationship between leguminous trees and neighbor diversity in tropical forests
Source: Commun Biol. 2020 Jun 19;3:317. doi: 10.1038/s42003-020-1041-y (PMC7305120; doi:10.1038/s42003-020-1041-y)
Supplement: Supplementary file 1 — Supplementary Information [file 42003_2020_1041_MOESM1_ESM.pdf]

Supplementary Figures

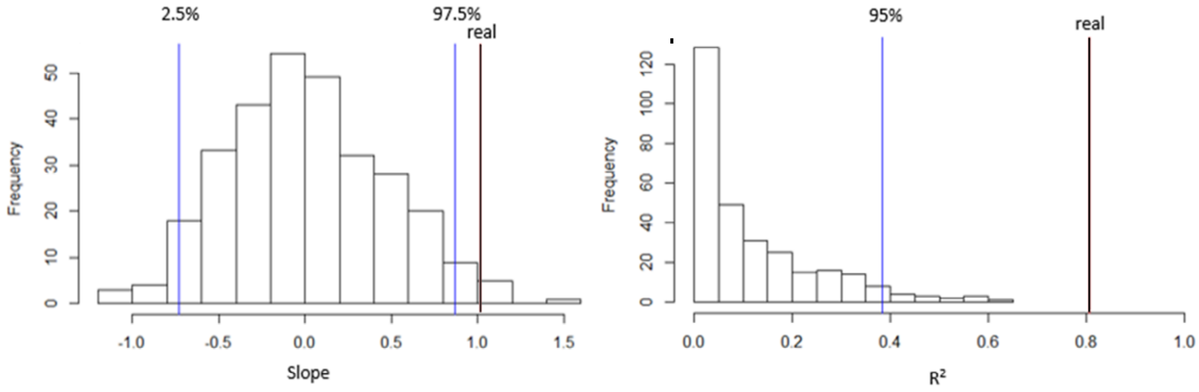

Supplementary Fig. 1 | Randomization test for the (**a**,) slope and **(b,)** R^2 of the relationship between proportion of legume species with positive *RND* and soil total N concentration of each forest plot. Blue lines indicate 2.5% and 97.5% quantile envelopes of the randomization test. Black line indicates the real value from the 11 plots. Method: Randomization was applied by choosing n non-legume species per plot, where n is the number of legume species in that plot. Proportion of chosen species with positive *RND* is calculated at a spatial radius of r = 2 m for each plot, and is plotted against the soil total N concentration and fitted by the linear model. This process is repeated 299 times and the envelope of slope (2.5% and 97.5%) and envelope of R^2 (95%) of the linear are compared with the real values from the 11 plots. Results showed that the real slope is outside the envelopes of randomized data between 2.5% and 97.5% for the slope, or larger than 95% for R^2.

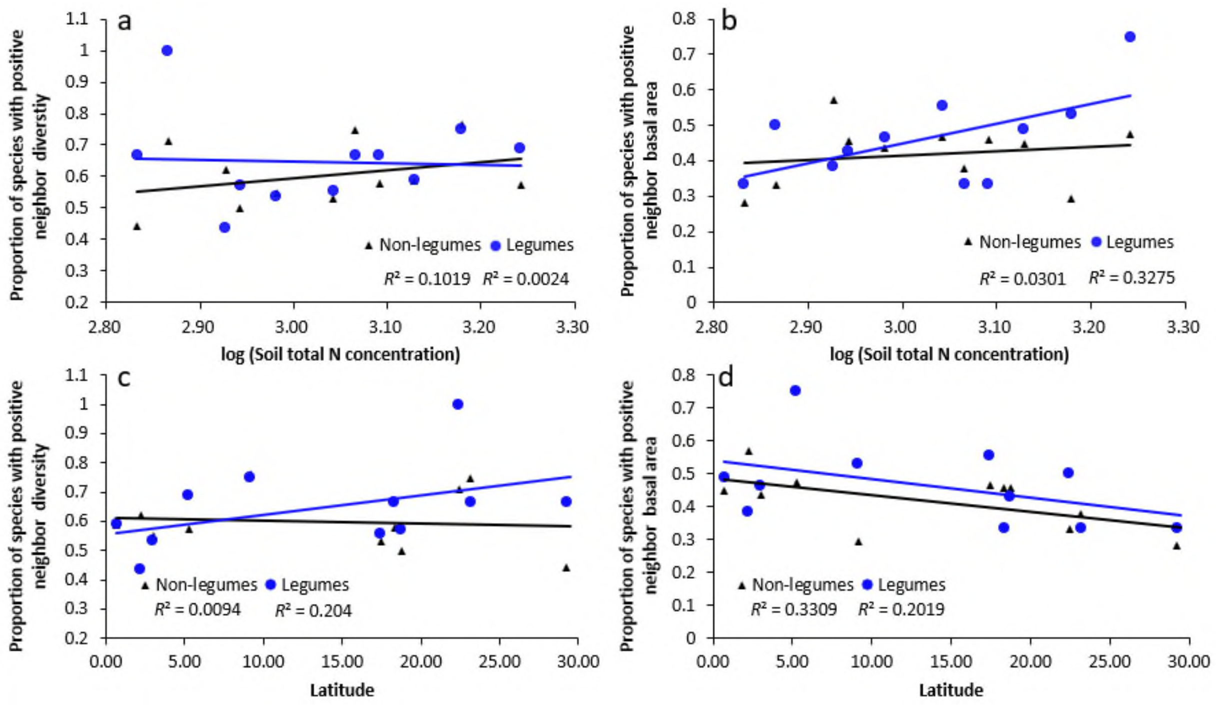

Supplementary Fig. 2 | Proportion of legume and non-legume species with positive neighbor diversity (*RND*) and positive neighbor basal area (*RNBA*) in relation to soil total N concentration (g m^{-3}) (**a, b)**, respectively; and in relation to latitude (**c, d)**, respectively, for 11 ForestGEO plots at a spatial radius of r = 4 m.

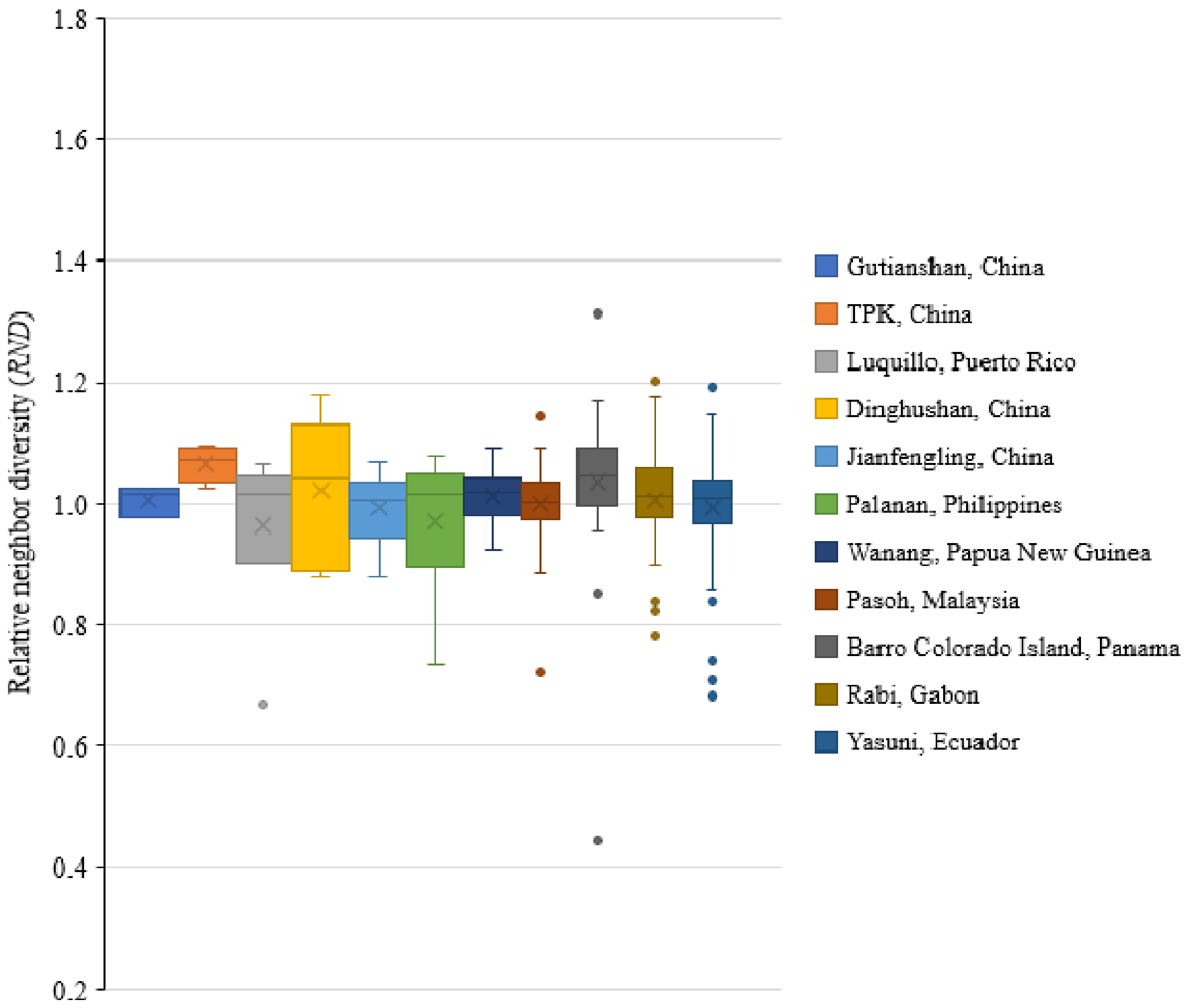

Supplementary Fig. 3 | Boxplot of legume relative neighbor density (*RND*, i.e., the ratio of the diversity of legume neighborhoods to the diversity of non-legume neighborhoods) at a 4 m radius from the focal legume for 11 ForestGEO plots displayed in order of number of legume species in each forest plot. n=3, 4, 6, 6, 7, 9, 16, 28, 32, 37 and 88 legume species for each plot, respectively.

Supplementary Tables

Supplementary Table 1 | Multiple regression analysis for the relationships among the proportion of legume species with positive relative neighbor diversity (*RND*), soil total N concentration, soil total P concentration, latitude, annual temperature and annual precipitation for 11 tropical forests.

[#]Proportion of legume species with positive *RND*	Estimate Std.	Error	*t* value	Pr(>|*t*|)
Intercept	-3.019e+00	9.562e-01	-3.157	**0.0252***
Soil total N concentration	1.155e+00	3.632e-01	3.180	**0.0245***
Soil total P concentration	1.570e-02	2.233e-01	0.070	0.9467
Latitude	-1.049e-04	4.510e-03	-0.023	0.9823
Annual temperature	6.565e-03	1.649e-02	0.398	0.7070
Annual precipitation	-6.607e-05	7.619e-05	-0.867	0.4255

#, with *p*-value: 0.0464 and R^2: 0.8400.

Supplementary Table 2 | Multiple regression analysis for the relationships among the proportion of legume species with positive relative neighbor basal area (*RNBA*), soil total N concentration, soil total P concentration, latitude, annual temperature, and annual precipitation for 11 tropical forests.

#Proportion of legume species with positive *RNBA*	Estimate Std.	Error	*t* value	Pr(>|*t*|)
Intercept	-2.645e+00	2.453e+00	-1.078	0.33
Soil total N concentration	6.596e-01	9.315e-01	0.708	0.511
Soil total P concentration	1.813e-01	5.728e-01	0.317	0.764
Latitude	4.377e-03	1.157e-02	0.378	0.721
Annual temperature	4.209e-02	4.230e-02	0.995	0.365
Annual precipitation	-1.700e-04	1.95e-04	-0.892	0.413

#, with *p*-value: 0.6213 and R^2: 0.4278.

Supplementary Table 3 | Soil total N and P concentration from the global ORNL DAAC database compared with those actual measured in three plots.

Plot name and country	Soil total N concentration for the soil depth interval 0-100 cm from the global ORNL DAAC database (g m^{-3})	Soil total P concentration for the soil depth interval 0-50 cm from the global ORNL DAAC database (g m^{-2})	Actual measured soil N concentration for the soil depth interval 0-10 cm (g/kg)	Actual measured soil P concentration for the soil depth interval 0-10 cm (g/kg)
Gutianshan, China	680.2	207.0	222.0±62.1	15.0±5.3
Jianfengling, China	877.1	485.2	203.5±36.1	12.6±1.8
Barro Colorado Island (BCI), Panama	1510.5	604.5	258.8±79.1	28.7±16.0

Supplementary Notes

Supplementary Note 1 | Acknowledgments to all persons, institutions and funds of eleven plots.

Jianfengling plot: The plot set up was supported by National Non-profit Institute Research Grant of CAF (RITFYWZX200902, CAFYBB2011004, CAFYBB2017ZE001, CAFYBB2017QC003), Public-welfare Forestry funds from State Forestry Administration of China (201104057) and maintenance funds of Jianfengling ecological station. The authors are grateful to Mingxian Lin, Jianhui Wu and many technicians for the logistic support, field assistance and other guidance.

Barro Colorado Island plot: The BCI forest dynamics research project was founded by S. P. Hubbell and R. B. Foster and is managed by R. Perez, S. Aguilar and S. Lao under the Forest Global Earth Observatory of the Smithsonian Tropical Research Institute in Panama.

Dinghushan plot: The plot set up and survey was supported by Strategic Priority Research Program of the Chinese Academy of Sciences (XDPB0203).

Gutianshan Plot: The plot set up and survey was supported by the NSFC (31470490).

Luquillo plot: The Luquillo Forest Dynamics Plot has been supported by grants BSR-8811902, DEB 9411973, DEB 0080538, DEB 0218039, DEB 0620910, DEB 0963447,

and DEB-1546686 from NSF to the Institute for Tropical Ecosystem Studies, University of Puerto Rico, and to the International Institute of Tropical Forestry USDA Forest Service, as part of the Luquillo Long-Term Ecological Research Program. The U.S. Forest Service (Dept. of Agriculture) and the University of Puerto Rico gave additional support.

Palanan plot: The 16-ha Palanan FDP was established cooperatively by the Institute of Biology at the University of the Philippines Diliman (UPD), Conservation International-Philippines, Isabela State University, and PLAN International with approval from Department of Environment and Natural Resources Region 2 and support from the Center for Tropical Forest Science. We particularly acknowledge the contributions of Perry S. Ong who was the Principal Investigator of the plot until his passing in March 2019.

Pasoh plot: Data from the Pasoh Research Forest was provided by the Forest Research Institute Malaysia-Forest Global Earth Observatory, Smithsonian Tropical Research Institute collaborative research project. Negeri Sembilan Forestry Department is the custodian of Pasoh Research Forest and we acknowledge the department for preserving the research forest.

Rabi plot: The Rabi 25-ha is a collaborative project of the National Center for Scientific and Technical Research (CENAREST) in Gabon, the Center for Conservation and Sustainability (CCS) of the Smithsonian Conservation Biology Institute (SCBI) and the Center for Tropical Forest Science - Forest Global Earth Observatories (CTFS-ForestGEO) of the Smithsonian

Tropical Research Institute. Funding for the first census was provided by Shell Gabon, CTFS-ForestGEO, and SCBI. Permission to conduct the field program in Gabon is provided by CENAREST. The plot is located in a conservation area of a forest concession of the Compagnie des Bois du Gabon (CBG). This is contribution #194 of the Gabon Biodiversity Program.

TPK plot: The plot is set up by Hongkong University and Kadoorie Farm and Botanic Garden, Hong Kong SAR, China. We thank many students and voluntary workers who attended the field investigation.

Wanang plot: The 50-ha Wanang Forest Dynamics Plot is a collaborative project of the New Guinea Binatang Research Center, the Center for Tropical Forest Science of the Smithsonian Tropical Research Institute, the Forest Research Institute of Papua New Guinea, the Czech Academy of Sciences and the University of Minnesota supported by NSF DEB- 1027297, NIH ICBG 5UO1TW006671, Swire & Sons Pty. Ltd. and Steamships Trading Co.. We acknowledge the government of Papua New Guinea and the customary landowners of Wanang for supporting and maintaining the plot.

Yasuni: We gratefully acknowledge the professional help of numerous biologists and field collaborators of the Yasuni forest dynamics plot, particularly Pablo Alvia and Milton Zambrano, who provided invaluable expertise on plant taxonomy. Pablo Sandoval, Karina Larco, Jairo Zambrano, Andrés Melo, Rosa Jiménez, Milton Zambrano, Jugalio Suárez,

Miguel García, Mayra Nacimba helped with fieldwork. Consuelo Hernández managed and improved the data quality. Pontificia Universidad Católica del Ecuador (PUCE) and STRI co-financed the first two censuses of the plot. PUCE offered financial support thought grant N-13373 to Renato Valencia. This study was endorsed by the Ministerio de Ambiente del Ecuador permits MAE-DPA0-2: No 004-2012-IC-FLO-MAE-DPO-2016-1542).
